# First-in-human study of intravenous bispecific CTLA-4 × OX40 antibody (ATOR-1015) in advanced solid malignancies☆

**DOI:** 10.1016/j.esmoop.2026.106068

**Published:** 2026-04-21

**Authors:** V. Ekström-Rydén, A. Carneiro, J. Yachnin, K. Staal Rohrberg, K. Enell Smith, P. Ellmark, R. Løvendahl Eefsen, G.J. Ullenhag

**Affiliations:** 1 Department of Immunology, Genetics and Pathology, Uppsala University, Uppsala, Sweden; 2 Department of Oncology, Uppsala University Hospital, Uppsala, Sweden; 3 Department of Oncology, Division of Haematology, Oncology and Radiation Physics, Skåne University Hospital Comprehensive Cancer Centre and Lund University, Lund, Sweden; 4 Department of Oncology, Karolinska University Hospital, Stockholm, Sweden; 5 Department of Oncology, Karolinska Institute, Stockholm, Sweden; 6 Department of Oncology, Copenhagen University Hospital—Rigshospitalet, Copenhagen; 7 Department of Clinical Medicine, Copenhagen University, Copenhagen, Denmark; 8 Alligator Bioscience AB, Medicon Village, Lund, Sweden; 9 Department of Immunotechnology, Lund University, Lund, Sweden; 10 Department of Oncology, Copenhagen University Hospital—Herlev and Gentofte, Copenhagen, Denmark

**Keywords:** bispecific antibody, metastatic cancer, phase I study, CTLA-4, OX-40

## Abstract

**Background:**

Ipilimumab, a monoclonal antibody against cytotoxic T-lymphocyte-associated protein 4 (CTLA-4), was the first approved checkpoint inhibitor; however, its use is hampered by treatment-related adverse events (TRAEs). ATOR-1015 is a bispecific antibody targeting CTLA-4 and OX40 designed to direct the effect to the tumor microenvironment and thereby improve the response rate and reduce TRAEs.

**Materials and methods:**

We conducted a phase I, first-in-human, multicenter study to determine the safety and tolerability of intravenously administered ATOR-1015 every second week. Secondary objectives were efficacy and pharmacokinetics. An exploratory objective was to investigate the pharmacodynamic effects on the immune system. Twenty-eight patients with advanced solid cancer were treated in the last line with ATOR-1015. A modified 3 + 3 dose-escalation design was applied.

**Results:**

The most common TRAEs were infusion-related reactions (IRRs) (*n* = 14, 50.0%), pyrexia (*n* = 3, 10.7%), myalgia (*n* = 3, 10.7%) and rash (*n* = 3, 10.7%). One serious AE occurred (grade III IRR). Best response (according to immune RECIST) was stable disease (*n* = 11, 39.3%), which lasted >4 months in six patients (21.4%). These patients showed a significant increase in inducible T-cell costimulator-expressing CD4+ and CD8+ central memory T cells 4 h after infusion. In all patients, temporary elevations in absolute lymphocyte count, B cells and T cells as well as interleukin-8, interferon-γ and tumor necrosis factor-α were observed.

**Conclusions:**

Although considered safe, the treatment induced neutralizing anti-drug antibodies in most patients, leading to IRRs and reduced efficacy due to low serum exposure, an effect that could not be overcome by increasing doses. Future development requires re-engineering the therapeutic format to minimize immunogenicity, thereby improving both safety and effectiveness.

## Highlights

• ATOR-1015 is a bispecific CTLA-4 × OX40 antibody designed to boost tumor response while limiting toxicity.
• In patients with mixed cancers, the most common AEs were IRR, pyrexia, myalgia and rash.
• Elevations in absolute lymphocyte counts, B and T cells, IL-8, IFN-γ and TNF-α were observed in the whole population.
• Best response (iRECIST) was SD (*n* = 11, 39.3%), which lasted >4 months in six patients (21.4%).
• Patients with SD >4 months had elevated ICOS-expressing CD4+ and CD8+ central memory T cells 4 h after infusion.

## Graphical abstract

**Figure:**
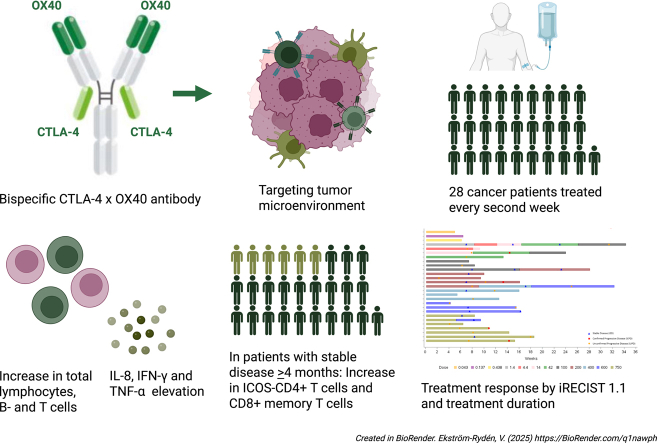

## Introduction

The immune system plays a key role in preventing cancer development, and established cancer has, by definition, evaded detection and elimination by the immune system.^1^ The discovery of checkpoint inhibitors (CPIs), i.e. cytotoxic T-lymphocyte-associated protein 4 (CTLA-4) and programmed death-(ligand) 1 blockers (PD-(L)1), has shown that the immune system has an intrinsic potential to overcome tumor immune resistance that may be unlocked when the immune cells are activated.

The CTLA-4 receptor is a suppressor of immune activation, which, in healthy individuals, functions to prevent autoimmunity and chronic inflammation.^2^ It is up-regulated on activated T cells and constitutively expressed on regulatory T cells (Tregs).^2^, ^3^, ^4^, ^5^ CTLA-4 reduces interleukin (IL)-2 production, suppresses T-cell proliferation and survival and hampers T-cell-mediated anticancer response.^6^ Up-regulation of CTLA-4 is a mechanism by which cancer cells evade detection and elimination by the immune system and it is overexpressed in the tumor microenvironment (TME).^3^^,^^4^^,^^7^ All mechanisms of CTLA-4-mediated immune regulation are not completely understood,^5^^,^^8^ but they are believed to be a combination of intrinsic and extrinsic functions.^5^ It is known that CTLA-4 outcompetes the costimulatory receptor CD28 by binding their common ligand B7 (CD80/86) with higher affinity,^3^^,^^5^ but there are other recognized functions of uncertain importance, such as intrinsic activation of Tregs^5^^,^^9^ and depletion of B7 from antigen-presenting cells by trogocytosis.^10^

Ipilimumab, which inhibits the CTLA-4 receptor, was the first approved CPI for the treatment of cancer.^11^ However, a major challenge with CTLA-4 blockers is that the systemic activation of T cells, which is necessary for their antitumor effects, can result in immune-related adverse events (IRAEs). These side-effects may be severe and require treatment discontinuation.^12^ Indeed, in clinical practice, it has been reported that ipilimumab monotherapy (3 mg/kg) causes grade 3-4 IRAEs in 27% of patients, and in combination with nivolumab in as many as 55%.^13^ The primary rationale for designing ATOR-1015 was to direct the CTLA-4 inhibition to the tumor site by using a bispecific antibody with simultaneous affinity to CTLA-4 and OX40, which is another target highly expressed in the TME.^14^ Such an approach could increase local drug exposure while decreasing systemic exposure and potentially reduce side-effects.^15^ OX40 has been identified as a promising novel drug target in cancer immunotherapy, and multiple OX40 agonists have been studied in a clinical setting.^16^^,^^17^ It belongs to the tumor necrosis factor (TNF) superfamily^18^ and works as a costimulatory molecule in antitumor immune response.^16^^,^^19^^,^^20^ OX40 is overexpressed on lymphocytes in the TME, particularly on activated T cells and Tregs,^7^^,^^21^ but it is only present at very low levels in peripheral blood.^22^ Stimulation of OX40 activates and promotes proliferation of CD4+^23^ and CD8+ T cells,^20^ and deactivates Tregs,^21^ resulting in facilitated tumor rejection in preclinical models.^19^ It has been hypothesized that a combination treatment with OX40 stimulation and a CTLA-4 inhibitor can result in a synergistic effect,^17^^,^^24^ and animal models have shown a favorable effect of combined treatment. Together, this supports the development of bispecific antibodies targeting both CTLA-4 and OX40, potentially improving therapeutic efficacy and safety over the monoclonal counterparts.^25^^,^^26^

Based on this, ATOR-1015, a bispecific antibody targeting CTLA-4 and OX40, was developed, aiming to (i) direct CTLA-4 targeting activity to the TME and (ii) realize the synergistic effects of combining OX40 agonism with CTLA-4 inhibition.^21^ ATOR-1015 is a bispecific antibody of human immunoglobulin G1 (IgG1) that inhibits CTLA-4 and induces OX40-mediated activity.^17^ The ultimate goal is to achieve a synergistic antitumor immune response through CTLA-4 inhibition/depletion and OX40 stimulation, with a decreased risk of systemic IRAEs by directing the activity to the TME.^17^ ATOR-1015 has been tested *in vitro* and *in vivo* in transgenic mice with human OX40 receptor (hOX40tg) carrying subcutaneous bladder (MB49) and colon cancer (MC38) tumors. ATOR-1015 was found to selectively bind tumor-infiltrating immune cells, activate and stimulate proliferation of CD8+ T cells in the TME, decrease tumor-infiltrating Tregs and reduce tumor growth.^17^ Overall, the preclinical data showed that ATOR-1015 has the potential to be a next-generation CTLA-4-targeting therapy that reduces toxicity and improves efficacy.

Based on these preclinical studies as well as safety and toxicology studies in non-human primates, we conducted a first-in-human phase I clinical trial. Safety and efficacy data are presented here, as well as the results from pharmacokinetic and pharmacodynamic analyses.

## Materials and methods

### Purpose

This clinical trial was a first-in-human, multicenter, open-label, phase I dose-escalation study of the intravenously administered bispecific antibody ATOR-1015 (NCT03782467). The primary objective was to determine the safety and toxicity profile of dose escalation, identifying the maximum tolerated dose (MTD), dose-limiting toxicities (DLTs) and a recommended phase II dose of ATOR-1015. The secondary objectives were to determine the clinical efficacy, pharmacokinetics and immunogenicity after repeated drug administrations. There was also an exploratory objective, which was to investigate the pharmacodynamic effect of ATOR-1015 on the immune system.

The study was approved by the central institutional review boards/independent ethics committees of the participating sites. This study was pre-registered (NCT03782467) and conducted in accordance with the Declaration of Helsinki (2013) and the European Medicines Agency ‘Guideline on Strategies to Identify and Mitigate Risks for First-in-Human Clinical Trials with Investigational Medicinal Products’ (doc. ref. EMEA/CHMP/SWP/28367/07 Rev 1). The study complied with the International Council for Harmonization (ICH) Guidelines for Good Clinical Practice and applicable regulatory requirements. Before study enrollment, written informed consent was obtained from each patient. Informed consent forms were in accordance with the guidelines of ICH.

### Patient recruitment

Included patients had a confirmed diagnosis of locally advanced (unresectable) or metastatic solid malignant disease, which was measurable according to immune RECIST (iRECIST)^27^ and who were refractory or intolerant to standard-of-care therapy. Other inclusion criteria were age ≥18 years, an Eastern Cooperative Oncology Group (ECOG) performance status of 0 or 1, a life expectancy of at least 3 months and acceptable blood laboratory values. Exclusion criteria were other anticancer medications within the previous 4 weeks, ongoing toxicity of grade ≥2 from another anticancer therapy and treatment with systemic immunosuppressants and corticosteroids equivalent to >10 mg prednisolone per day. Full inclusion, exclusion and withdrawal criteria can be found in Supplementary Material S1, available at https://doi.org/10.1016/j.esmoop.2026.106068.

### Study procedures

This study was designed to include 12 flat-dose levels: 0.043 mg, 0.137 mg, 0.438 mg, 1.4 mg, 4.4 mg, 14 mg, 42 mg, 100 mg, 200 mg, 400 mg, 600 mg and 750 mg. A minimum anticipated biological effect level (MABEL)-based approach was used for selecting the start dose. The study used an accelerated dose-escalation design followed by a ‘modified 3 + 3 design’. Treatment started in single-patient cohorts for dose levels <100 mg. In the event of a grade ≥2 toxicity with a duration >72 h, or two or more grade ≥2 toxicities, an additional two patients were recruited to the same dose-level cohort, and the cohort sizes for the subsequent increased dose levels were expanded to contain at least three patients per dose step. Escalation of the dose was allowed following the first two treatment cycles, at the discretion of the Dose Review Committee (DRC) or sponsor, up to a dose level that had been declared safe by the DRC.

**Figure 1:**
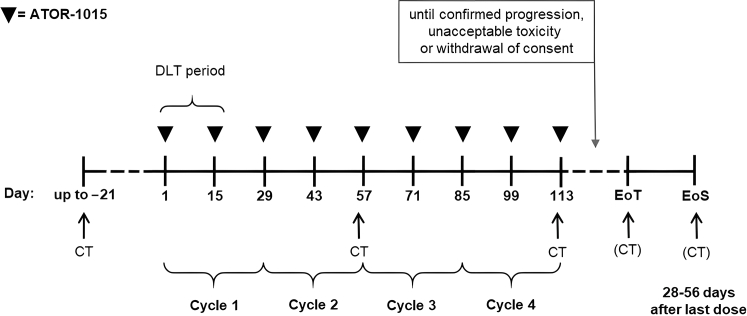
**Timeline showing dosage of ATOR-1015 and key assessments.** CT, computed tomography; DLT, dose-limiting toxicity; EoS, end of study visit; EoT, end of treatment visit.

After a screening period of up to 21 days, treatment was initiated with intravenous administration of ATOR-1015 every 14 days (day 1 and day 15 in cycles of 28 days). A schedule visualizing dosage and key assessments is illustrated in Figure 1.

The safety of ATOR-1015 was continuously evaluated through regular physician appointments including medical history, clinical examination, vital parameters, electrocardiogram, laboratory tests and documentation of any suspected adverse events (AEs). AEs were classified using the Common Terminology Criteria for Adverse Events (CTCAE) version 5.0, and the relation to the study drug was assessed. AEs were monitored until 28 days after the last treatment and regularly followed thereafter. AEs of special interest (AESIs) in this study were pre-defined as grade ≥2 IRR, grade ≥2 cytokine release syndrome and grade ≥2 IRAEs.

DLTs were evaluated in the timeframe between the first dose of ATOR-1015 [cycle 1 day 1 (C1D1)] and 48 h after the second dose (C1D15). A list of toxicities pre-determined to be regarded as DLTs are presented in Supplementary Material S2, available at https://doi.org/10.1016/j.esmoop.2026.106068. All DLTs were to be regarded as ATOR-1015-related, except in cases where a clearly documented alternative explanation for the AE could be identified. MTD was defined as one dose step below the level where DLTs occurred in two or more patients. The frequency of DLTs at the MTD could, by definition, not exceed one in six patients.

Radiological evaluation with computed tomography scan and assessment according to iRECIST were carried out at baseline and every 8 weeks starting at the end of cycle 2. Clinical efficacy was measured as the frequency of objective response, disease control rate, best overall response and duration of response. Full definitions of the terms used in response evaluation are listed in Supplementary Material S3, available at https://doi.org/10.1016/j.esmoop.2026.106068.

For pharmacokinetic analyses, peripheral blood from the arm contralateral to the infusion was drawn at specific time points (Supplementary Material S1, available at https://doi.org/10.1016/j.esmoop.2026.106068) and analyzed at Charles River Laboratories, UK. Pharmacokinetic parameters were derived from individual serum concentration data and calculated with Phoenix (WinNonlin) pharmacokinetic software, version 1.4 (Certara, Radnor, PA, Build No. 6.4.0.768), using a non-compartmental approach suitable for the intravenous route of administration. The pharmacokinetic parameters assessed included *C*_max_, *T*_max_, half-life (*t*_½_) and area under the curve.

For the assessment of immunogenicity of ATOR-1015, anti-drug antibody (ADA) titers in serum were measured at pre-determined time points (Supplementary Table S1, available at https://doi.org/10.1016/j.esmoop.2026.106068). The samples were analyzed in a screening assay, and positive samples in a confirmatory assay. The neutralizing capacity of ADAs was also assessed using an enzyme-linked immunosorbent assay-based method. Pharmacodynamic evaluation was carried out at specific time points through blood sampling for cytokines and immune phenotyping, including B-cell, T-cell and natural killer (NK) cell populations and their activation status (full description in Supplementary Table S1 and Supplementary Material S4, available at https://doi.org/10.1016/j.esmoop.2026.106068).

Treatment was withdrawn in case of radiological progressive disease (PD), unacceptable toxicity, apparent clinical deterioration of the patient’s condition or fulfillment of any other withdrawal criteria. Patients could continue treatment with ATOR-1015 up to 2 years after the last recruited patient’s first dose. There was no pre-determined maximum number of doses.

### Statistical analysis

Categorical variables were presented as numbers and, if appropriate, percentages. Continuous variables were presented as *n*, mean, median, standard deviation and range (minimum and maximum) as appropriate.

Changes in white blood cell counts, levels of cytokines and T-cell activation status over time were assessed and compared with the temporal changes in biomarkers between individuals with lasting immune-related stable disease (iSD) (>4 months) and patients with shorter iSD or iPD. To do so, a generalized least squares (GLS) analysis was carried out for each biomarker to investigate the changes in biomarker values over repeated measurements. The GLS model was used to account for the correlation between repeated measures within the same individual by specifying an autoregressive [AR(1)] correlation structure. A *P* value <0.05 (two-sided) was considered statistically significant. Missing observations were excluded from the GLS analyses (complete-case approach). No multiple-comparison adjustments were applied to the *P* values.

The model included:

• Fixed effects: To estimate the average effect of each time point and the interaction between time and response to treatment on the biomarker values.
• Correlation structure: An AR(1) correlation structure to account for the correlation between measurements taken at different times within the same individual.

All statistical analyses were carried out in R version 4.3.3 (R Foundation for Statistical Computing, Austria).

## Results

### Demographics

Between 2019 and 2021, 29 patients were enrolled, of whom 28 were treated with ATOR-1015. One patient did not receive the study drug due to accelerated disease progression and clinical deterioration before cycle 1. The treated cohort consisted of 14 males (50%) and 14 females (50%), with a median age of 55 years (range 40-72 years). The most common diagnoses were colorectal cancer (*n* = 11, 39.3%), ovarian cancer (*n* = 3, 10.7%), pancreatic cancer (*n* = 3, 10.7%) and uveal malignant melanoma (*n* = 3, 10.7%). Initial ECOG performance status was 0 in 15 patients (53.6%) and 1 in 13 patients (46.4%). All 28 treated patients (100%) had received previous chemotherapy. A majority (57.1%) had received four or more lines of chemotherapy treatment. Eighteen patients (64.3%) had received previous immunotherapy. For full details of baseline patient characteristics, see Table 1.

**Table 1:** Patient characteristics

Patient characteristics	
Age, years	
Mean	55.6
Range	40-72
Sex, *n* (%)	
Male	14 (50)
Female	14 (50)
ECOG performance status, *n* (%)	
0	15 (53.6)
1	13 (46.4)
Prior chemotherapy, *n* (%)	28 (100)
One regimen	4 (14.3)
Two regimens	1 (3.6)
Three regimens	7 (25.0)
Four or more regimens	16 (57.1)
Other prior cancer therapies, *n* (%)	
Immunotherapy	18 (64.3)
Radiotherapy	10 (35.7)
Various therapies	18 (64.3)
Diagnosis, *n* (%)	
Colorectal cancer	11 (39.3)
Ovarian cancer	3 (10.7)
High-grade serous adenocarcinoma	1 (3.6)
Low-grade serous adenocarcinoma	1 (3.6)
Unspecified adenocarcinoma	1 (3.6)
Pancreatic cancer	3 (10.7)
Uveal malignant melanoma	3 (10.7)
Cancer of the gall-bladder or bile ducts	2 (7.1)
Cervical cancer (cervix uteri)	1 (3.6)
Cutaneous malignant melanoma	1 (3.6)
Sarcomatoid mesothelioma of the pleura	1 (3.6)
Lung squamous-cell carcinoma	1 (3.6)
Cardiac cancer (stomach)	1 (3.6)
Undifferentiated carcinoma of the ethmoid sinuses	1 (3.6)

Characteristics of patients (*n* = 28) treated with ATOR-1015, presented as numbers and frequency in percentage within parentheses.

Seven patients started ATOR-1015 treatment with doses between 0.043 and 42 mg. Four patients started at 100 mg, four at 200 mg, three at 400 mg, three at 600 mg and seven at 750 mg. Six patients underwent intrapatient dose escalation. The patients received a median of 2.5 treatment cycles (five doses) (range 1-8 cycles) over a median of 53.5 days (range 1-250 days). The most common reasons for eventual study withdrawal were clinical disease progression (*n* = 9, 32.1%), clinical deterioration (*n* = 7, 25%) and confirmed disease progression (*n* = 5, 17%).

### Safety

Treatment-related AEs (TRAEs) were reported in 17 (60.7%) patients. The most common (experienced by ≥10%) were infusion-related reaction (IRR) (*n* = 14, 50.0%), pyrexia (*n* = 3, 10.7%), myalgia (*n* = 3, 10.7%) and rash (*n* = 3, 10.7%). Half of the treated patients (*n* = 14, 50%) presented with an AESI which was grade ≥2 IRR. Ten of the IRRs were reported as grade 2, and four as grade 3, which was the highest observed toxicity grade of any AE. Grade 4-5 events did not occur. Cytokine release syndrome or IRAEs were not observed in any patient during the trial.

**Figure 2:**
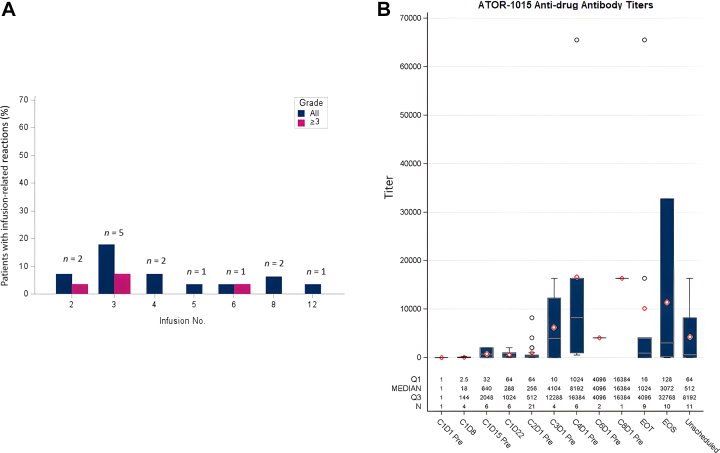
(A) Percentage of patients with infusion-related reactions (IRRs) by grade and number of infusions. The *y*-axis shows the percentage of patients in the treated population of 28 patients. The *x*-axis shows the number of infusions with ATOR-1015. Above each bar, the number of affected patients is displayed. The dark blue bars show all affected patients at that treatment point. When grade 3 IRRs occurred, a second pink bar to the right displays the affected subset of patients. In total, 14 patients (50%) experienced an IRR at any time point. (B) Anti-drug antibody (ADA) titers presented over time during treatment with ATOR-1015. All patients who received at least one dose and had an ADA sample collected both before and during treatment were included (*n* = 25). The titers are presented as dilution factor. The term ‘pre’ denotes that the sample was drawn before dosing on the corresponding day. Unscheduled visits were aggregated into the right-most column. The shaded box spans the first- to third-quartile titer values. The median value for a timepoint is represented by a horizontal line and the mean value by a diamond. Individual outlier values are shown as circles. C, cycle; D, day; EoS, end of study visit; EoT, end of treatment visit.

IRRs were not observed in any patient at the first infusion; all reactions debuted between infusion number 2 and 12. As the number of infusions increased, more patients presented with their first IRR. All IRRs occurred during the infusions or up to 30 min after. The timing of the IRRs according to treatment cycle is illustrated in Figure 2A. The durations of IRR symptoms were short (hours) and the reactions were reversible with limited interventions (intravenous fluid, antihistamines and corticosteroids). In most cases, ATOR-1015 could be restarted on the same day at a lower rate of infusion without recurrence of IRR symptoms. In two cases of grade 3 IRR, the study drug was discontinued. Further treatments proceeded as planned in the other patients, although eight of them experienced repeated IRRs. Of note, there were no observed IRRs at the lowest dose levels (0.043-42 mg) and the severity of IRR symptoms and the extent of required interventions increased with higher doses. At ATOR-1015 doses of 100, 200, 400, 600 and 750 mg, the rate of IRRs observed was 75%, 50%, 33%, 67% and 86%, respectively. The four grade 3 IRRs were reported at the highest dose levels [400 mg (*n* = 1), 600 mg (*n* = 1) and 750 mg (*n* = 2)]. One of the grade 3 IRRs was registered as a serious AE (SAE) and DLT and occurred at the 750 mg dose level. The patient experienced general discomfort and a decrease in blood pressure and oxygen saturation, and was treated with antihistamines, corticosteroids and fluids. The patient was fully recovered after 45 min. No other ATOR-1015-related SAEs or DLTs occurred during the trial.

ATOR-1015 was discontinued due to TRAEs in three cases (10.7%), including two grade 3 IRRs at the 750 mg dose level and one general deterioration of physical health at the 100 mg dose level. Ten study patients died within 8 weeks of end of study, of whom five died within 4 weeks of their last dose of ATOR-1015. All deaths were caused by disease progression. All TRAEs are listed in full in Table 2.

**Table 2:** Summary of ATOR-1015 TRAEs

ATOR-1015 TRAEs	*n* (%)
Number of patients who received treatment	28 (100.0)
Number of patients with TRAEs	17 (60.7)
Infusion-related reaction	14 (50.0)
Myalgia	3 (10.7)
Pyrexia	3 (10.7)
Rash	3 (10.7)
Dizziness	2 (7.1)
Fatigue	2 (7.1)
Flushing	1 (3.6)
Abdominal pain	1 (3.6)
Arthralgia	1 (3.6)
Chest discomfort	1 (3.6)
Chills	1 (3.6)
Cortisol increased	1 (3.6)
Diarrhea	1 (3.6)
Dry eye	1 (3.6)
Free thyroxine increased	1 (3.6)
Headache	1 (3.6)
Hypokalemia	1 (3.6)
Nausea	1 (3.6)
Non-cardiac chest pain	1 (3.6)
Throat irritation	1 (3.6)
Vitiligo	1 (3.6)
Back pain	1 (3.6)

A summary of all TRAEs (treatment-emergent adverse events judged by the investigator as related to ATOR-1015) is displayed. The number of patients affected and frequency are presented. The analysis set comprises the 28 patients who received treatment.

### Efficacy

**Figure 3:**
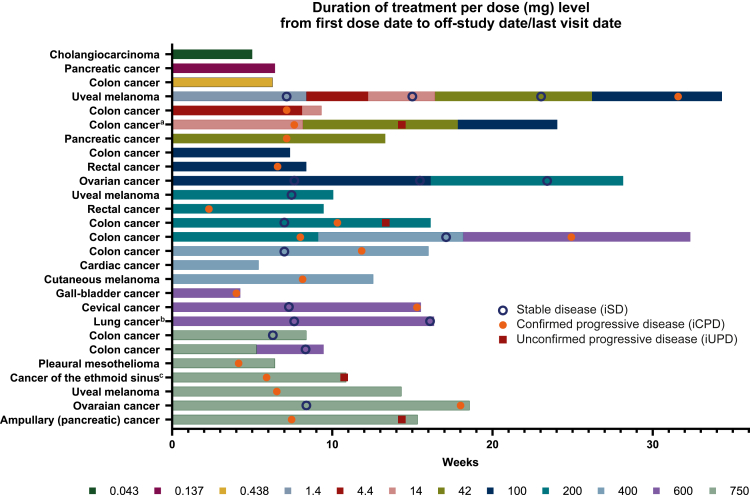
**Duration of treatment and patients’ responses.** Swimmer plot showing the duration of treatment per dose level with patient’s response over time. The 27 individuals displayed are all patients with pre-treatment measurable disease per immune RECIST (iRECIST) who had at least one radiographic assessment after treatment, or discontinued study medication early due to disease progression. Hence, one patient who did not receive treatment and one who discontinued the study before the first radiological evaluation due to general physical health deterioration are not displayed. ^a^The patient continued study treatment after immune-related progressive disease (iPD) due to an ambiguous radiology report at week 15, which initially was interpreted as immune-related stable disease (iSD). The imaging results were then re-evaluated, iPD was confirmed, and the patient was taken off study treatment. ^b^Squamous-cell carcinoma of the lung. ^c^Undifferentiated carcinoma of the ethmoid sinus.

In the intention-to treat population (*n* = 29), no patients experienced an objective response according to iRECIST. Best overall response was iSD for at least 2 months in 11 patients (37.9%), of which 6 (20.7%) had iSD for >4 months. All cases of iSD were observed at doses of 14 mg or higher, except for one patient treated at the 1.4 mg dose step. There were eight patients (27.6%) with immune-related unconfirmed PD (iUPD) and three patients (10.3%) with immune-related confirmed PD. One patient did not receive any treatment (3.4%) and six patients (20.6%) were not assessable due to clinical deterioration that led to study withdrawal before the first planned radiology assessment. The overall disease control rate for at least 2 months (immune complete response + immune partial response + iSD) was 37.9%. In the swimmer plot (Figure 3), individual patient diagnoses, treatment doses and responses to treatment over time are illustrated. Supplementary Table S2, available at https://doi.org/10.1016/j.esmoop.2026.106068, presents patients’ treatment responses grouped by diagnoses.

### Pharmacokinetics and immunogenicity

Patients who were treated at least once with ATOR-1015 and underwent post-treatment pharmacokinetic measurements were included in the pharmacokinetic analysis set (*n* = 28). Following administration of ATOR-1015 14 mg or higher, the peak concentrations in serum were measurable and observed directly after the end of infusion. Serum concentrations were generally higher after the first and second administration, compared with the same timepoints in later cycles. The highest concentration was observed after the first dose in cycle 1.

Elimination followed a bi-phasic course. Mean clearance decreased as the dose increased, and the time of the last measurable concentration in serum (*t*_last_) tended to occur later with higher doses. Pharmacokinetic results are presented in detail in Supplementary Table S3, available at https://doi.org/10.1016/j.esmoop.2026.106068.

The ADA analysis set consisted of 25 patients (patients who were treated at least once with ATOR-1015, and had a pre- and post-treatment immunogenicity measurement). All patients tested negative for ADAs before treatment start on day 1, except one individual in whom the result was interpreted as false positive. At day 8, more than half of the patients tested positive for ADAs, and from day 15 onward, all but one patient had developed ADAs with drug-neutralizing capacity. This result is consistent with the decreased serum concentrations of ATOR-1015 observed after repeated administrations. The only patient who did not develop ADAs was treated in the 750 mg dose cohort. In this patient, ATOR-1015 half-life was longer and systemic exposure was greater compared with the rest of the dose cohort. The patient suffered from undifferentiated carcinoma of the ethmoidal sinus and experienced iUPD at the first radiological evaluation. ADA titers over time for all patients are illustrated in Figure 2B.

### Exploratory pharmacodynamics

After the first dose of ATOR-1015, absolute lymphocyte counts and the levels of B and T cells (CD3+, CD4+ and CD8+) were elevated in the whole population at 4 h post-infusion [14.3%, 9.2%, 20.0%, 20.0% and 16.7% higher in median, respectively (Supplementary Figure S1, available at https://doi.org/10.1016/j.esmoop.2026.106068)]. The concentrations of IL-1β, IL-2, IL-4, IL-10, IL-12p70 and IL-13 were either undetectable or very low, while those of IL-6, IL-8, interferon (IFN)-γ and TNF-α were measurable. At different time points, IL-8, IFN-γ and TNF-α were temporarily increased [in median: IFN-γ +10.2% at 4 h, TNF-α +11.8% at day 15 and IL-8 +13.1% at day 3, +8.6% at day 8 and +12.1% at day 15 (Supplementary Figure S2, available at https://doi.org/10.1016/j.esmoop.2026.106068)]. Otherwise, the changes in white blood cells and cytokines were small and did not vary significantly over time. No dose–response relationship could be established (Supplementary Figure S3 and S4, available at https://doi.org/10.1016/j.esmoop.2026.106068). When separating patients with and without lasting iSD >4 months, there was a statistically significant increase in inducible T-cell co-stimulator (ICOS)-expressing CD4+ T cells (39.0%, *P* = 0.044) and CD8+ memory T (TCM) cells (40.4%, *P* = 0.030) at 4 h post-infusion in patients with iSD lasting >4 months (Supplementary Figure S5, available at https://doi.org/10.1016/j.esmoop.2026.106068). No other group-by-time interactions reached statistical significance.

## Discussion

CTLA-4 inhibitors have been demonstrated to be effective, but are associated with toxicities that limit their clinical utility. Thus, development of novel CTLA-4-targeting therapies with reduced toxicity and enhanced efficacy is warranted. In this phase I first-in-human study, we treated 28 cancer patients in the last line with CTLA-4–OX40 dual-targeting bispecific antibody ATOR-1015.

Treatment was concluded to be safe at investigated doses (up to 750 mg) and the MTD was not reached. The most notable TRAE was grade 2-3 IRR, which occurred more frequently after repeated doses. These reactions could reasonably be attributed to the immunogenicity of ATOR-1015 with ADA formation, as both ADA and IRRs increased with the number of treatment cycles. All grade 3 events occurred at higher dose levels, and the severity of symptoms and the extent of necessary interventions increased with the dosage. The only reported SAE and DLT was one grade 3 IRR. The IRRs were of short duration and successfully treated with minor interventions such as corticosteroids, antihistamines and fluid support. Patients experiencing IRRs were monitored more frequently post-infusion and symptomatic treatment was given as needed. Due to the occurrence of IRRs, premedication was initially considered and later made mandatory from the second dose onward (after a DRC decision). The premedication included paracetamol (acetaminophen) and an antihistamine for all patients. For patients who had experienced an IRR, an intravenous glucocorticoid was also administered before all subsequent doses of ATOR-1015 (e.g. prednisolone 50-100 mg).

Eight patients experienced IRRs that resolved without corticosteroid treatment. The other six patients with IRRs were treated with corticosteroids, of which four were taken off-study due to clinical disease progression (*n* = 3) or clinical deterioration (*n* = 1) before receiving further ATOR-1015 treatment. The remaining two steroid-treated patients received corticosteroid premedication before all subsequent infusions, but both developed new IRRs nevertheless. Of note, both were treated at the highest (750 mg) dose step. One of the patients experienced one more IRR and then continued with further ATOR-1015 treatment without any IRRs. The other patient experienced two repeated IRRs and was taken off-study since the last IRR was considered as unacceptable toxicity (the study’s only DLT). There was no indication that corticosteroid premedication would prevent or milden IRRs, but it is difficult to draw any conclusions due to the low number of patients who received corticosteroid premedication.

Although the corticosteroids used as premedication or treatment for IRRs may negatively influence the efficacy of ATOR-1015, this is highly speculative. Despite differences in pharmacokinetics compared with ATOR-1015, the negligible impact of corticosteroids on the activity of ipilimumab^28^ indicates that the lack of efficacy of ATOR-1015 can most likely not be attributed to the steroids administered to some patients.

During immunotherapy, especially with T-cell engagers, cytokine release syndrome is usually described as a challenge^29^ and for bispecific antibodies the frequency tends to be particularly high. Cytokine release syndrome has been reported in 50%-80% for teclistamab,^30^ glofitamab,^31^ talquetamab^32^ and erlanatamab.^33^ For CPIs, the occurrence is much lower: ∼1% in monotherapy^34^ and 3.5% during combination therapy.^35^ Cytokine release syndrome was expected to be a possible AE for ATOR-1015, but it was not observed in this small study population despite the occurrence of IRRs and presence of ADAs.

No objective responses were observed within the study. The best response to treatment was iSD, which was observed more frequently in patients who received ATOR-1015 doses of 100 mg or higher. iSD was observed in patients with colon cancer, uveal melanoma, lung cancer (squamous epithelial) and cervical cancer. Most benefit was observed in the two assessable patients with ovarian cancer: one patient (with high-grade serous adenocarcinoma) received 100 mg and experienced iSD during the entire study period of 28 months, and the other (with unspecified adenocarcinoma) received 750 mg until week 18 (followed by iUPD). Five out of nine patients with colon cancer showed iSD at least once, all of which were observed at doses of 200 mg or higher. The results could indicate a benefit in ovarian and colon cancer, particularly at higher doses, but a larger study with more patients per diagnosis and dose group would be needed to confirm these results. It is noteworthy that the patients included in this study were heavily pretreated with other anticancer medications, and one could speculate that multiple previous lines of treatment could have negatively impacted their susceptibility to CTLA-4 inhibition.

ADAs were observed in all but one patient after repeated doses. *In silico* immunogenicity prediction showed low risk for immunogenicity of the compound; however, bispecific antibodies are considered more prone to generation of ADAs compared with monospecific antibodies.^36^ For ATOR-1015, the ADAs were likely directed to the CTLA-4 binding domain, which is derived from the IgV domain of human CD86 and contains five-point mutations and a complex glycosylation pattern; however, the specificity of the ADA was not confirmed experimentally. As a comparison, ADAs have been described also following ipilimumab therapy, but to a much lower extent (0%-26% of patients in different studies).^37^ ADAs had an impact on drug exposure after repeated doses of ATOR-1015 and are likely related to the high incidence of IRRs in the study. Further, ATOR-1015 had a significantly shorter half-life and exposure compared with ipilimumab.^38^ A *post hoc* analysis found that the degree of sialylation on the CTLA-4 binding part of ATOR-1015 was related to serum half-life, and the low level of sialylation of ATOR-1015 may have impacted the pharmacokinetic properties. In summary, the exposure achieved in this study may not have been sufficient for the assessment of the clinical activity of the target combination.

Absolute leukocyte counts and levels of B cells, T cells and IFN-γ were significantly increased at 4 h and TNF-α at day 15 after the first dose of ATOR-1015. For IL-8 we observed a consecutive increase starting from day 3 after the first dose and at the subsequent test points on days 8 and 15. IL-8 can have both tumor-suppressive and tumor-promoting effects. IL-8 induces chemotaxis and activation of granulocytes, stimulates phagocytosis and promotes angiogenesis.^39^ It also stimulates the release of hematopoietic cells from the bone marrow into the circulation.^39^ IL-8 is also believed to increase tumoral resistance to cytotoxic immune cells and promote metastatic formation.^39^ In patients who experienced SD over time (≥4 months), an increase in ICOS-expressing CD4+ T cells (39%, *P* = 0.044) and CD8+ TCM cells (40.4%, *P* = 0.030) was observed at 4 h after ATOR-1015 infusion.

Established CTLA-4 inhibitors are effective in some but not all patients with cancer. They also have a problematic toxicity profile with frequent IRAEs. A desire to optimize CTLA-4 inhibitors with increased efficacy and/or decreased toxicity has been recognized by many, and there is much ongoing research in this field. In addition to bispecific antibodies, other approaches to optimizing CTLA-4-inhibiting treatments have recently been described. One example is botensilimab, a CTLA-4-inhibiting antibody containing an Fc with enhanced affinity to Fc-gamma receptors (FcγR) on NK cells, monocytes/macrophages and other immune cells to strengthen T-cell activity, deplete intratumoral Tregs and promote activation of antigen-presenting cells.^40^ In a population with different CPI-refractory solid cancers treated with botensilimab, the SD rate was 38%, which is similar to that observed with ATOR-1015; in contrast, botensilimab showed objective responses in 13.3%.^40^ In light of these recent findings, a potential way to develop ATOR-1015 further could be Fc-region enhancement to bind FcγRs with higher affinity.

In conclusion, ATOR-1015 was considered safe at the investigated dose levels and temporary elevations in absolute lymphocyte counts, B cells and T cells as well as IFN-γ, TNF-α and IL-8 were observed after the first dose. Individuals with lasting iSD showed a significant increase in ICOS-expressing CD4+ T cells and CD8+ TCM cells at 4 h after infusion. Unfortunately, ADAs were detected in a majority of patients and were found to have a drug-neutralizing capacity. Once ADAs developed, IRRs occurred. Further, higher dose levels could not overcome the reduction in exposure caused by the ADA to increase efficacy. While we believe that the target combination explored in this study is promising, a new bispecific construct would need to be developed to enable higher exposure and lower ADAs and IRRs. The CTLA-4-binding domain used in the ATOR-1015 molecule is likely not suitable for further clinical development. A bispecific construct based on an appended IgG, such as the bispecific antibody format described by Nyesiga et al.,^41^ would maintain bivalent binding to both CTLA-4 and OX40 and potentially overcome the poor tumor exposure due to the high ADA levels observed in this study. The present study contributes to the general knowledge of bispecific antibodies by enhancing the understanding of possibilities and limitations that bispecific antibodies may have in future cancer therapies.
