# Supplementary material for: First-in-human study of intravenous bispecific CTLA-4 × OX40 antibody (ATOR-1015) in advanced solid malignancies
Source: ESMO Open. 2026 Apr 21;11(5):106068. doi: 10.1016/j.esmoop.2026.106068 (PMC13123599; doi:10.1016/j.esmoop.2026.106068)
Supplement: Supplemental Material [file mmc1.pdf]

# Supplementary Materials

**Supplementary Material S1 - Full inclusion and exclusion critera, and withdrawal criteria**

Inclusion Criteria

In order to participate in this study, a patient had to meet all of the following inclusion criteria:

1. Provided written informed consent.
2. Was ≥18 years of age at the time of signing the informed consent form.
3. Had a body weight ≥40 kg.
4. Had a diagnosis of advanced and/or refractory solid malignancy (histologically or cytologically documented) that was metastatic or unresectable and had received standard of care therapy and remaining therapeutic options were participation in a clinical study or best supportive care.
5. Had an Eastern Cooperative Oncology Group (ECOG) performance status of 0 or 1.
6. Had a minimum of one measurable tumor lesion ≥10 mm in diameter (≥15 mm for nodal lesions) per iRECIST.
7. Had a minimum of one lesion ≥1 cm in a non-irradiated area.
8. Had a life expectancy of at least 3 months.
9. Had acceptable hematologic laboratory values defined as:

a) Neutrophils ≥1.5 × 109/L, without growth factor stimulation within 3 weeks prior to the blood test

b) Platelets ≥100 × 109/L

c) Hemoglobin ≥5.9 mmol/L (~95 g/L), without transfusion or erythropoietin therapy within 4 weeks prior to the blood test

10. Had acceptable clinical chemistry laboratory values defined as:

a) Albumin ≥24 g/L

b) Creatinine ≤1.5 × upper limit of normal (ULN) or glomerular filtration rate of ≥45 mL/min

c) AST ≤3 × ULN without, and ≤5 × ULN with hepatic metastasis/metastases

d) ALT ≤3 × ULN without, and ≤5 × ULN with hepatic metastasis/metastases

e) Total bilirubin ≤1.5 × ULN (except patients with Gilbert's syndrome, who had to have total bilirubin <3.0 mg/dL)

11. For women of childbearing potential: Had a negative highly sensitive serum ( $\beta$ -human chorionic gonadotropin) pregnancy test at screening.
12. Was willing to comply with all study procedures.

Exclusion Criteria

Patients could not participate in the clinical study if they met any of the following exclusion criteria:

1. Had received anti-cancer medications within 4 weeks prior to first dose (6 weeks required for nitrosourea or mitomycin) except for medications with half-lives <5.5 days.
Bisphosphonates, denosumab and androgen deprivation therapies such as luteinizing hormone releasing hormone (gonadotropin-releasing hormone) agonists were allowed if patients were stabilized on treatment for at least 4 weeks prior to screening.
2. Had not recovered from AEs to at least Grade 1 by CTCAE version 5.0 due to prior anti-cancer medications (except for alopecia, Grade 2 neuropathy, or adequately controlled Grade 2 endocrinopathies) prior to signing the ICF.
3. Had received radiotherapy within 14 days before first dose (palliative radiotherapy for pain allowed).
4. Had symptomatic, steroid-dependent or progressive brain metastasis/metastases within 4 weeks prior to signing the ICF.
5. Had clinically significant cardiac disease, including:
a) Known congestive heart failure Grade III or IV by the New York Heart Failure Association
b) Myocardial infarction within 6 months prior to signing the ICF
c) Onset of unstable angina within 6 months prior to signing the ICF
6. Was an organ transplant recipient.
7. Had a history of another primary malignancy, except for:
a) Malignancy treated with curative intent and with no known active disease within 5 years prior to first dose of ATOR-1015
b) Adequately treated non-invasive basal skin cancer or squamous cell skin carcinoma
c) Adequately treated uterine cervical cancer stage 1B or less
8. Had an autoimmune disorder requiring immune modulating treatment during the last 2 years prior to first dose of ATOR-1015. Patients with vitiligo, resolved atopy, limited psoriasis, hypothyroidism stable on hormone replacement, type I diabetes, Grave's or Hashimoto's disease under treatment and stable were allowed.

9. Was receiving treatment with systemic immunosuppressant medication (inhaled and low dose systemic corticosteroids, i.e., ≤10 mg prednisolone, or equivalent, per day was allowed for at least 4 weeks prior to first dose of ATOR-1015).

10. Had a known positive serology for human immunodeficiency virus.

11. Had a positive serology for hepatitis B (anti-HBc) or known prior hepatitis B infection.

12. Had a positive serology for hepatitis C (anti-HCV) (unless undetectable virus load by polymerase chain reaction after HCV treatment or due to immunoglobulin therapy).

13. Was participating, or had participated within the previous 4 weeks, in an investigational drug or device study with any intervention.

14. Had been exposed to live or live attenuated vaccine within 4 weeks prior to signing informed consent form.

15. Was a female patient who was pregnant, nursing, or, of childbearing potential, not willing to use a highly effective form of contraception during treatment and for at least 6 months after the last dose of ATOR-1015.

Highly effective forms of contraception included (if using hormonal contraception this method had to be supplemented with a barrier method, preferably male condom):

- Combined (estrogen and progestogen containing) hormonal contraception associated with
- inhibition of ovulation
- Progestogen-only hormonal contraception associated with inhibition of ovulation
- Bilateral tubal occlusion
- Vasectomized partner
- Abstinence defined as when this was in line with the preferred and usual lifestyle of the patient

16. Was a sexually active male patient with a female partner not practicing effective double barrier contraceptive methods or not willing to abstain from sperm donation during the study and for 6 months after last dose of ATOR-1015.

17. Any condition that, in the opinion of the Investigator, would have placed the patient at increased risk or precluded the patient's compliance with the study.

Removal of Patients from Therapy or Assessment

A patient could be withdrawn from the study for any of the following reasons:

- Withdrawal of consent. The patients could, at any time, discontinue their participation in the study
- Confirmed progressive disease (iCPD) according to iRECIST

• Clear clinical deterioration where continued study treatment would not be in the best interest of the patient, as judged by the Investigator

• Unacceptable toxicity

• Unrelated AE

• The patient's general condition contraindicated continued study treatment, as judged by the Investigator

• Permanent discontinuation of ATOR-1015 treatment (for any reason)

• The patient started any prohibited medication

• Delay in dosing for more than 8 weeks due to toxicity

• Non-compliance with the Clinical Study Protocol as judged by the Investigator and/or Sponsor

• Pregnancy

• The patient was lost to follow-up

• The study was terminated by the Sqponsor

**Supplementary Material S2 - Dose-limiting toxicities**

The following toxicities were pre-determined to be regarded as dose-limiting toxicities (DLTs):

- Grade 4 IRR, or Grade 3 IRR not resolving to a lower grade within 24 hours.
- Grade 4 elevation of aspartate aminotransferase (AST), alanine aminotransferase (ALT) or bilirubin.
- >7 days duration of grade 3 elevation of AST, ALT or bilirubin
- >7 days duration of grade 4 neutropenia or thrombocytopenia.
- Any Grade ≥3 non-hematologic toxicity, except for laboratory deviations without clinical consequence, that resolved to grade ≤2 within 14 days.

**Supplementary Material S3 - Definition of response evaluation**

Clinical efficacy was measured as the frequency of objective responses (immune complete response [iCR] or partial response [iPR]), disease control rate (iCR + iPR + immune stable disease [iSD]), best overall response (BOR), duration of response, and duration of stable disease. Best overall response was categorized into iCR, iPR, iSD, unconfirmed progressive disease (iUPD) and confirmed progressive disease (iCPD). Duration of response was defined as time from documentation of iCR or iPR to disease progression according to iRECIST or death. In patients with confirmed iSD according to

iRECIST, duration of iSD was defined as time from treatment initiation to disease progression according to iRECIST or death.

**Supplementary Table S1**

Blood sampling and analyzes at specific time points

Blood samples for pharmacokinetic, pharmacodynamic and immunogenic evaluation were drawn during the first three cycles, before the dose was administered (pre-treatment) and at specific time points thereafter. Pharmacodynamic sampling included immunophenotyping and blood cytokines. ATOR-1015 was administered at day 1 and 14 of each cycle.

| Treatment number | Pre-treatment | 5 minutes | 1 hour | 4 hours | 8 hours | 24 hours (day 2) | 48 hours (day 3) | 8 days |
|---|---|---|---|---|---|---|---|---|
| **Cycle 1 treatment 1** | PK ADA PD | PK PD | PK | PK PD | PK | PK PD | PK PD | PK ADA PD |
| **Cycle 1 treatment 2** | PK ADA | PK | PK | PK PD | | | | PK ADA |
| **Cycle 2 and 3, treatment 1** | PK ADA | PK | PK | PK PD | | | | |
| **Cycle 2 and 3, treatment 2** | PK ADA | | | | | | | |
| **Cycle 4 and onward, treatment 1** | ADA | | | | | | | |
| **Unscheduled visit** | PK, ADA, PD | | | | | | | |
| **End-of treatment visit** | PK, ADA, PD | | | | | | | |
| **End-of study visit** | PK, ADA | | | | | | | |

*Abbreviations: PK, Pharmacokinetic sampling. ADA, anti-drug antibody sampling. PD, Pharmacodynamic sampling.*

**Supplementary Material S4**

Description of pharmacodynamic analyzes

Cytokine levels, lymphocyte counts and T-cell phenotypes and activation markers were analyzed at Cerba Research. Cytokine levels in serum was analyzed using MSD and a pre-validated 10-plex Pro-

inflammatory kit. The following cytokines were included: IL-1β, IL-2, IL-4, IL-6, IL-8, IL-10, IL-12p70, IL-13, TNF-α and IFN-γ. Lymphocyte counts were analyzed using a pre-validated T cell, B cell, NK cell immunophenotyping and enumeration panel. Cells were stained with antibodies against CD45, CD3, CD4, CD8, CD16:CD56, CD19 and a viability marker and analyzed by flow cytometry. Analyses were performed in BD Truecount™ tubes to allow phenotyping with absolute cell counts. T-cell phenotypes and activation markers were analyzed using custom-made validated flow cytometry panels. Cells were stained with antibodies against CD45, CD3, CD4, CD8, CD25, CD127, CCR7, CD45RA, ICOS, Ki67, eomesodermin and a viability marker.

**Supplementary Table S2: Study patient's diagnoses, starting doses of ATOR-1015, and treatment responses**

The table displays treatment responses according to cancer diagnose, and how large proportion of patients with the same diagnose achieved a certain response to ATOR-1015 treatment. Values are presented as number of patients and frequency in percent. Treatment response was defined according to iRECIST.

| **Diagnosis** | **n** | **Starting doses in milligrams** | **iSD n (%)** | **iUPD n (%)** | **iCPD n (%)** | **Not evaluable n (%)** |
|---|---|---|---|---|---|---|
| Colon cancer | 9 | 0.438, 4.4, 14, 100, 200, 200, 400, 750, 750 | 5 (55) | 1 (11) | 1 (11) | 2 (22) |
| Rectal cancer | 2 | 100, 200 | 0 (0) | 2 (100) | 0 (0) | 0 (0) |
| Uveal melanoma | 3 | 1.4, 200, 750 | 2 (67) | 1 (33) | 0 (0) | 0 (0) |
| Ovarian cancer | 3 | 100, 100, 750 | 2 (67) | 0 (0) | 0 (0) | 1 (33) |
| Cervical cancer | 1 | 600 | 1 (100) | 0 (0) | 0 (0) | 0 (0) |
| Pancreatic cancer | 3 | 0.137, 42, 750 | 0 (0) | 1 (33) | 1 (33) | 1 (33) |
| Lung cancer (squamous epithelial) | 1 | 600 | 1 (100) | 0 (0) | 0 (0) | 0 (0) |
| Cholangiocarcinoma (gall bladder and bile ducts) | 2 | 0.43, 600 | 0 (0) | 1 (50) | 0 (0) | 1 (50) |
| Pleura | 1 | 750 | 0 (0) | 1 (100) | 0 (0) | 0 (0) |
| Cutaneous melanoma | 1 | 400 | 0 (0) | 1 (100) | 0 (0) | 0 (0) |
| Stomach (Cardia) | 1 | 400 | 0 (0) | 0 (0) | 0 (0) | 1 (100) |
| Head-and-neck (ethmoidal sinus) | 1 | 750 | 0 (0) | 0 (0) | 1 (100) | 0 (0) |

*Abbreviations: iCR, immune complete response. iCPD, immune confirmed progressive disease. iRECIST, immune Response Evaluation Criteria in Solid Tumors. iSD, immune stable disease. iUPD, immune unconfirmed progressive disease.*

**Supplementary Table S3 – pharmacokinetic results**

A summary of the pharmacokinetic test results from Cycle 1 grouped by dose cohort.

| Cycle 1 Day 1 | | | | | | | | | | |
|---|---|---|---|---|---|---|---|---|---|---|
| Target Dose (mg) | n | Mean $T_{max}$ (h) | Mean $C_{max}$ (ng/mL) | Mean $C_{max}$/D (ng/mL) /(mg) | Mean $AUCt_{last}$ (ng h/mL) | Mean $AUCt_{last}$/D (ng h/mL) /(mg) | Mean $t_{1/2}$ (h) | Mean $V_z$ (mL) | Mean CL (mL/h) | Mean $t_{last}$ (h) |
| 0.043 | 1 | BLQ | BLQ | BLQ | BLQ | BLQ | BLQ | BLQ | BLQ | BLQ |
| 0.137 | 1 | BLQ | BLQ | BLQ | BLQ | BLQ | BLQ | BLQ | BLQ | BLQ |
| 0.438 | 1 | 2.03 | 39.7 | 90.7 | ND | ND | ND | ND | ND | 3.03 |
| 1.4 | 1 | 2.08 | 88.0 | 62.8 | 256 | 183 | NC | NC | NC | 6.00 |
| 4.4 | 1 | BLQ | BLQ | BLQ | BLQ | BLQ | BLQ | BLQ | BLQ | BLQ |
| 14 | 1 | 2.08 | 1280 | 91.3 | 9480 | 617 | 11.8 | 23800 | 1400 | 47.1 |
| 42 | 1 | 2.10 | 2830 | 67.4 | 18900 | 451 | 14.7 | 42900 | 2020 | 49.7 |
| 100 | 4 | 2.18 | 8760 | 87.6 | 61900 | 619 | 22.5 | 48900 | 1630 | 78.8 |
| 200 | 4 | 2.48 | 25000 | 125 | 201000 | 1010 | 35.5 | 48800 | 1130 | 145 |
| 400 | 3 | 2.51 | 53500 | 134 | 440000 | 1100 | 44.5 | 56200 | 910 | 153 |
| 600 | 3 | 2.74 | 88300 | 147 | 657000 | 1090 | 31.1 | 38100 | 871 | 128 |
| 750 | 6 | 2.70 | 120000 | 160 | 897000 | 1200 | 35.5 | 47400 | 989 | 224 |
| **Cycle 1 day 15** | | | | | | | | | | |
| Target Dose (mg) | n | Mean $T_{max}$ (h) | Mean $C_{max}$ (ng/mL) | Mean $C_{max}$/D (ng/mL) /(mg) | Mean $AUCt_{last}$ (ng h/mL) | Mean $AUCt_{last}$ /D (ng/mL) /(mg) | | | | Mean $t_{last}$ (h) |
| 0.043 | 1 | BLQ | BLQ | BLQ | BLQ | BLQ | | | | BLQ |
| 0.137 | 1 | BLQ | BLQ | BLQ | BLQ | BLQ | | | | BLQ |
| 0.438 | 1 | 2.08 | 34.0 | 77.7 | ND | ND | | | | 2.08 |
| 1.4 | 1 | 2.08 | 63.3 | 45.2 | 205 | 147 | | | | 6.00 |
| 4.4 | 1 | 2.08 | 277 | 63.0 | 763 | 173 | | | | 5.50 |
| 14 | 1 | 2.05 | 469 | 33.5 | 1070 | 76.5 | | | | 5.53 |
| 42 | 1 | 2.10 | 1940 | 46.1 | 6030 | 144 | | | | 6.02 |
| 100 | 4 | 2.65 | 6970 | 69.7 | 160000 | 1600 | | | | 45.9 |
| 200 | 3 | 5.79 | 12700 | 63.4 | 51800 | 259 | | | | 8.57 |
| 400 | 3 | 2.58 | 53800 | 134 | 176000 | 440 | | | | 6.38 |
| 600 | 3 | 3.33 | 82100 | 137 | 282000 | 470 | | | | 6.98 |
| 750 | 6 | 3.67 | 87600 | 117 | 812000 | 1090 | | | | 48.4 |

*Abbreviations:*
*BLQ, below limit of quantification*
*$C_{max}$, the maximum observed concentration measured after dosing. $C_{max}$/D, the $C_{max}$ divided by the administered dose.*
*$AUCt_{last}$, the area under the concentration versus time curve from the start of dose administration to the last observed quantifiable concentration, using the linear trapezoidal method.*
*$AUCt_{last}$ /D, the $AUCt_{last}$ divided by the administered dose.*
*$t_{1/2}$, the apparent terminal elimination half-life.*
*$V_z$, volume of distribution based on the terminal elimination phase after intravenous dose.*
*CL, total body clearance after intravenous dose.*
*$T_{last}$, the time of the last quantifiable concentration.*

**Supplementary Figure S1**

**White blood cell levels in blood after ATOR-1015 treatment**

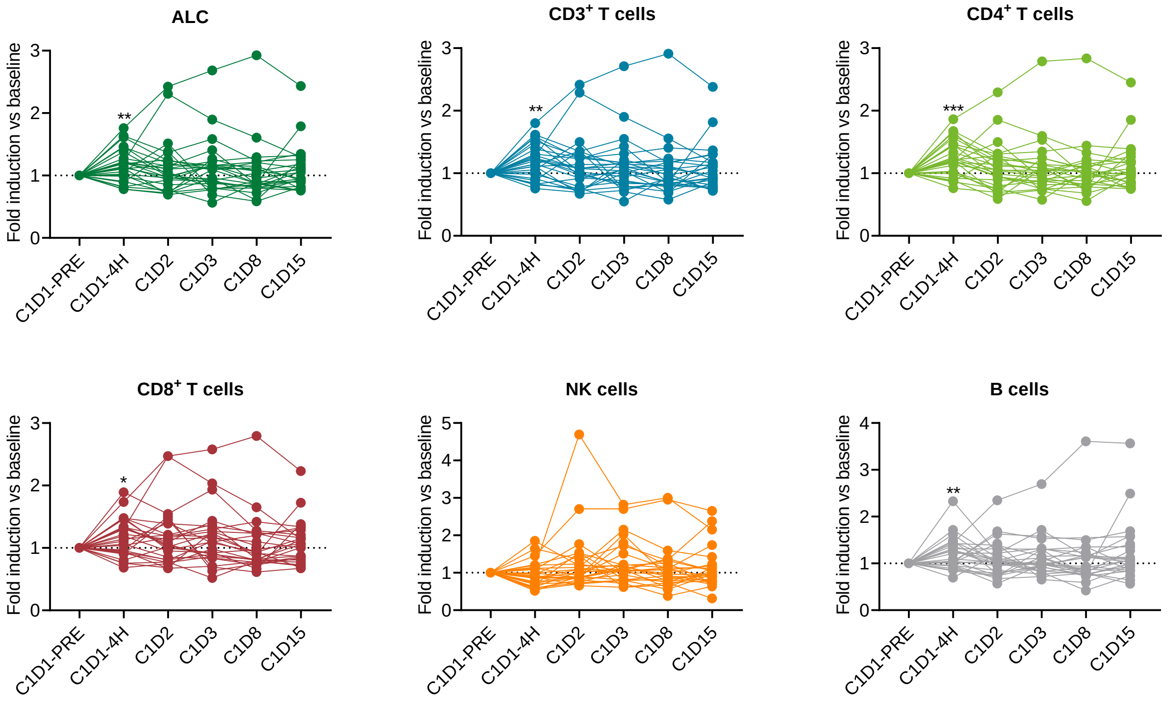


*Number of lymphocytes during the first cycle relative to baseline. Absolute lymphocyte counts (ALC) and the numbers of CD3+, CD4+, and CD8+ T cells, NK cells, and B cells are presented as fold induction over baseline. Data from each patient are shown, and statistical differences compared to baseline were analyzed using the Wilcoxon matched-pairs signed rank test (*, p<0.05; **, p<0.01; ***, p<0.001).*

**Supplementary Figure S2**

**Cytokine levels in serum after ATOR-1015 treatment**

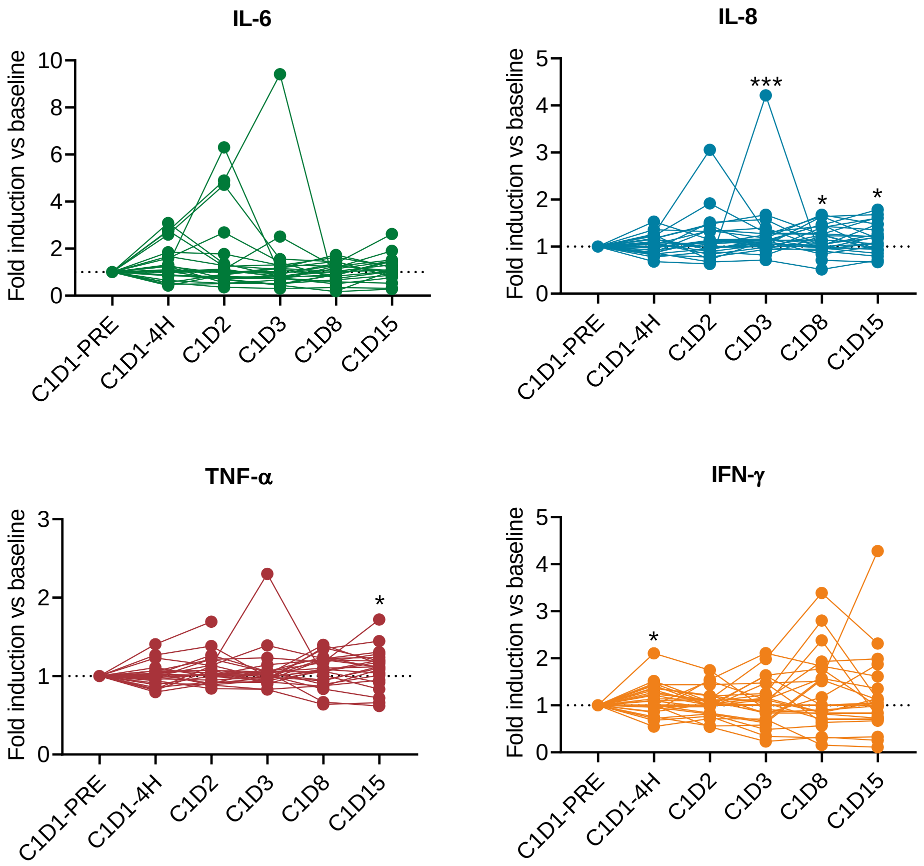


*Cytokine release in serum during the first cycle relative to baseline. The levels of IL-6, IL-8, TNF-α, and IFN-γ in serum are presented as fold induction over baseline. Data from each patient are displayed, and statistical differences compared to baseline were analyzed using the Wilcoxon matched-pairs signed rank test (*, p<0.05; ***, p<0.001). The dotted line represents baseline. The levels of IL-1β, IL-2, IL-4, IL-6, IL-10, IL-12p70 and IL-13 were very low or undetectable, and are thereby not displayed. Time points: CD1-PRE = cycle 1, day 1, pre-treatment. C1D1-4H = four hours post-infusion. C1D2 = cycle 1 day 2. C1D3 = cycle 1 day 3, C1D8 = cycle 1 day 8. C1D15 = cycle 1 day 15.*

## Supplementary Figure S3

## White blood cell levels in blood after treatment in relation to dose of ATOR-1015

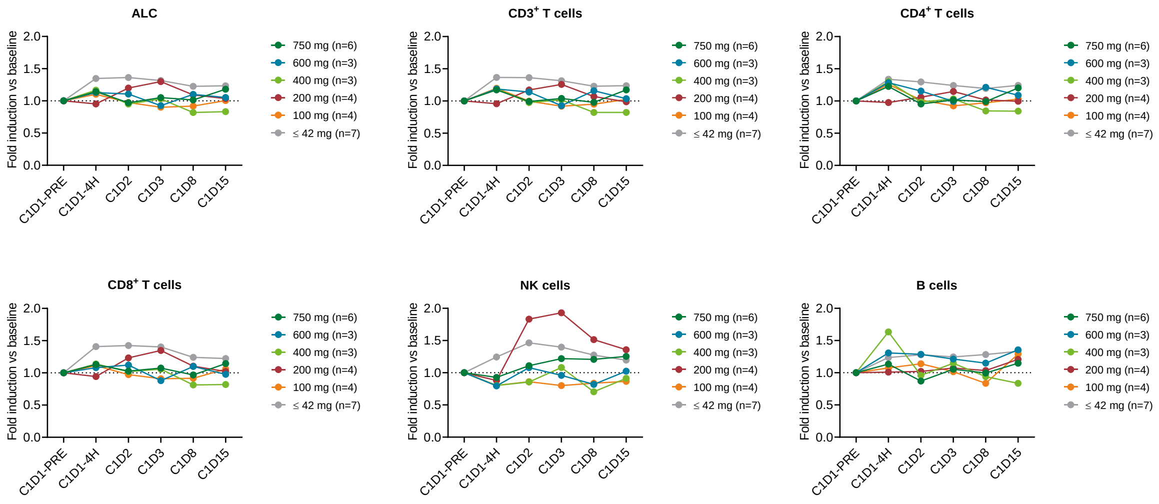


*Dose-response relationship for the number of lymphocytes in peripheral blood. Absolute lymphocyte counts (ALC) and the numbers of CD3+, CD4+, and CD8+ T cells, NK cells, and B cells following different fixed doses of ATOR-1015 are shown. Data are presented as the mean fold induction over baseline for each dose cohort.*

## Supplementary Figure S4

## Cytokine levels in serum after treatment in relation to dose of ATOR-1015

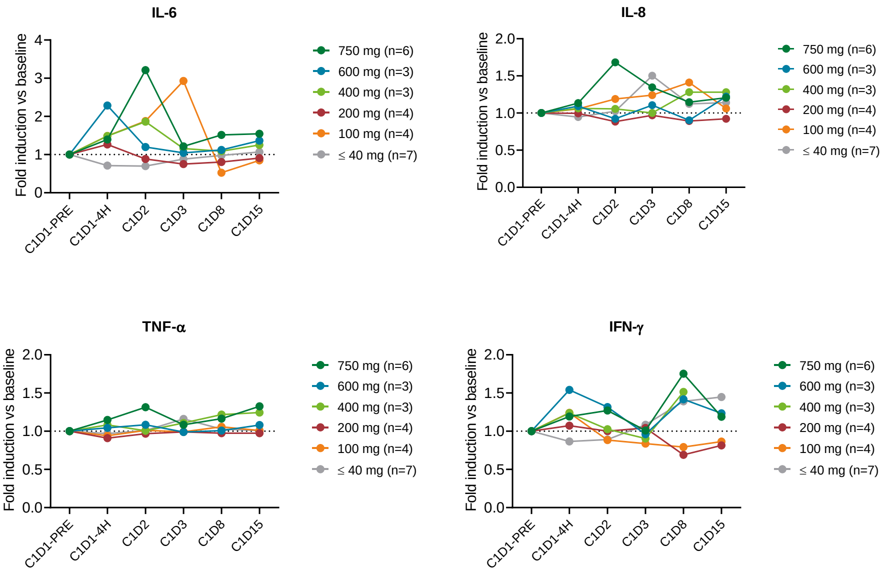


*Dose-response relationship for cytokine release in serum. The levels of IL-6, IL-8, TNF-α, and IFN-γ in serum following different fixed doses of ATOR-1015 are shown. Data are presented as the mean fold induction over baseline for each dose cohort.*

**Supplementary Figure S5**

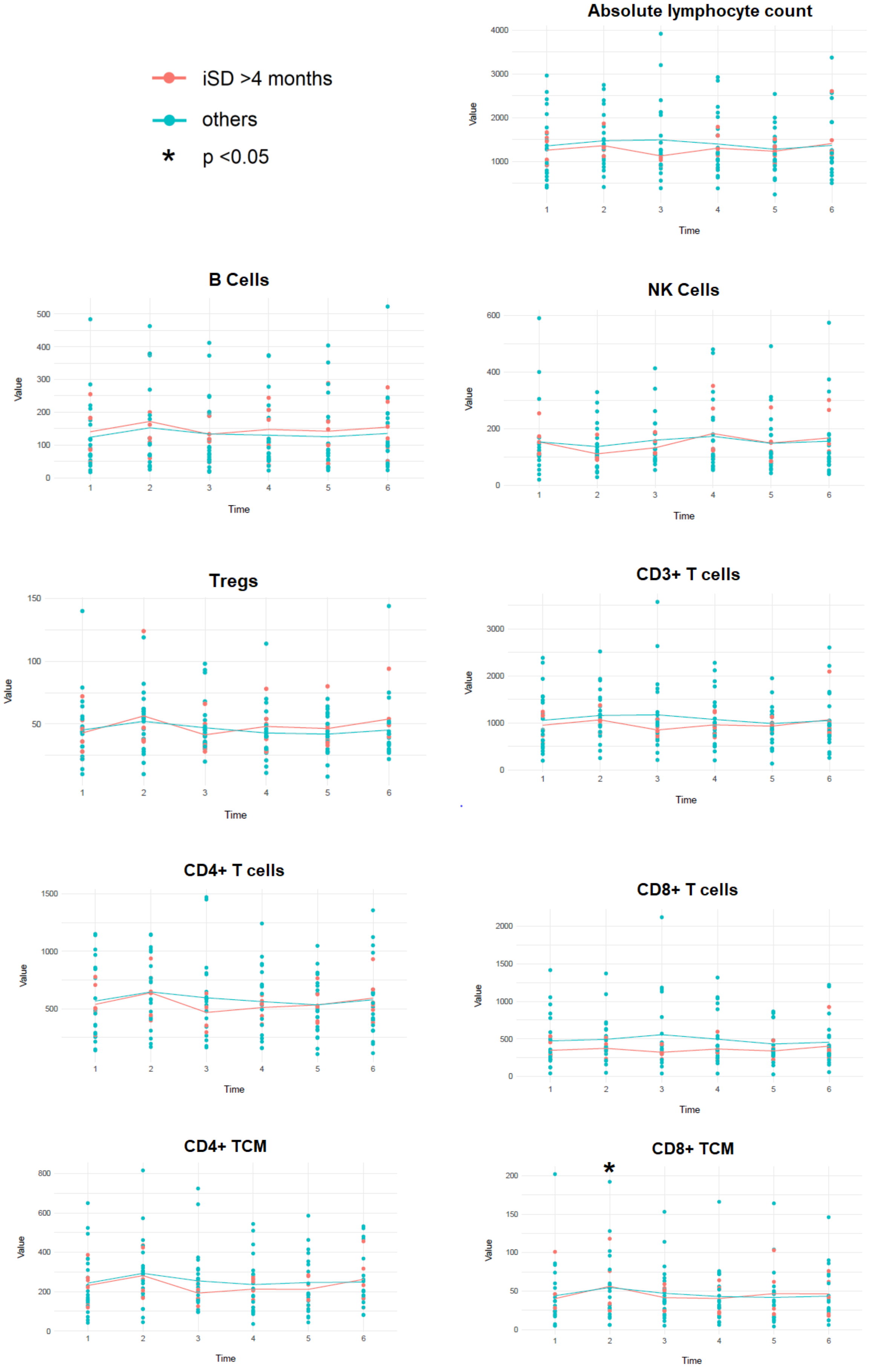

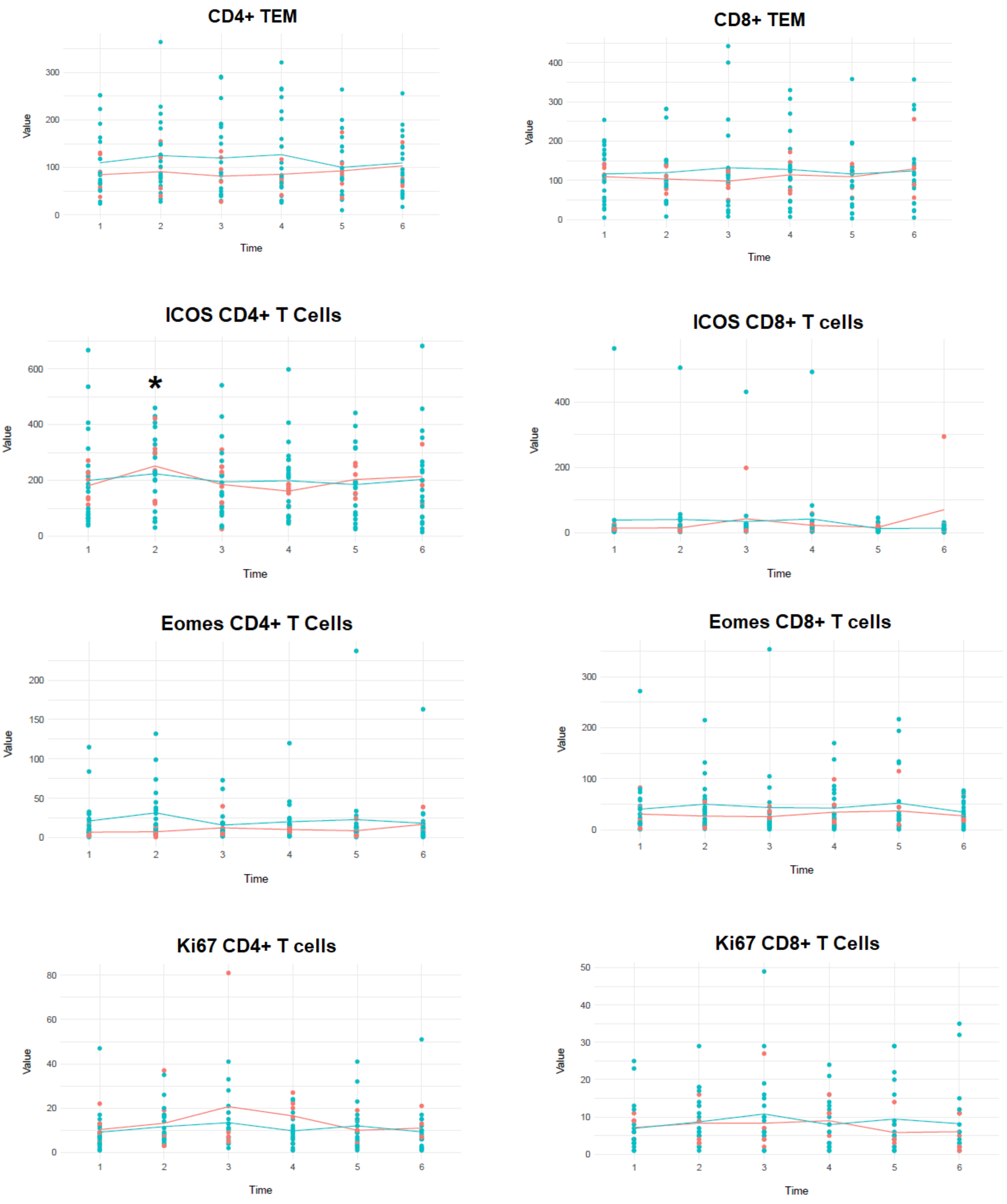


*Changes in immune cell populations 15 days after the first treatment of ATOR-1015. Patients are displayed in two groups according to treatment response: stable disease (iSD) according to iRECIST1.1 lasting 4 months or longer (orange), compared to pall other patients (blue). * marks a p-value below 0.05.*
